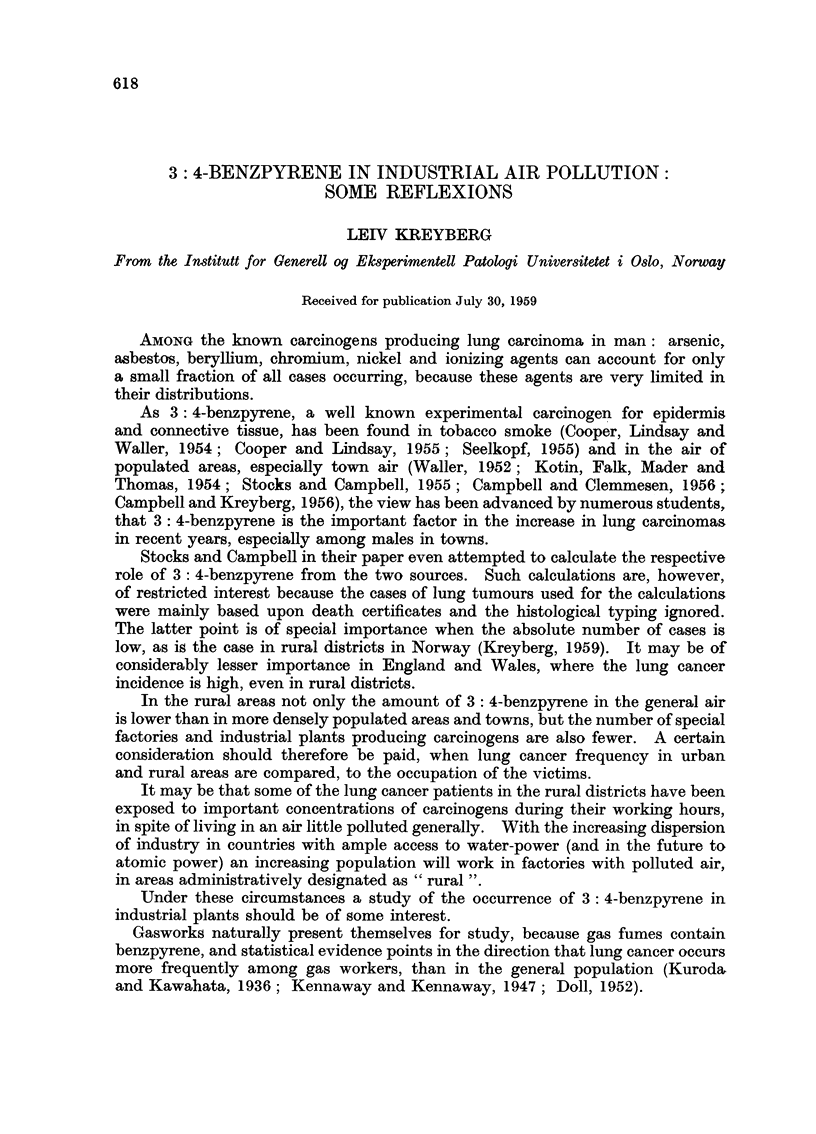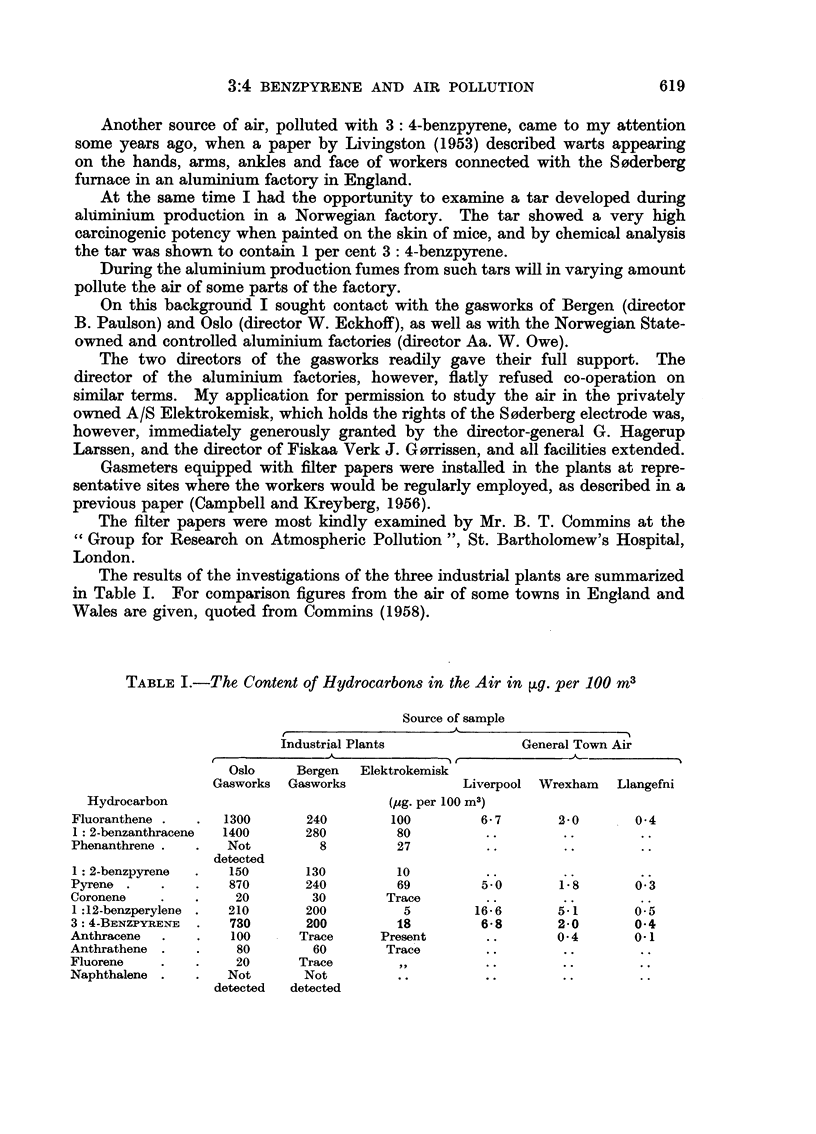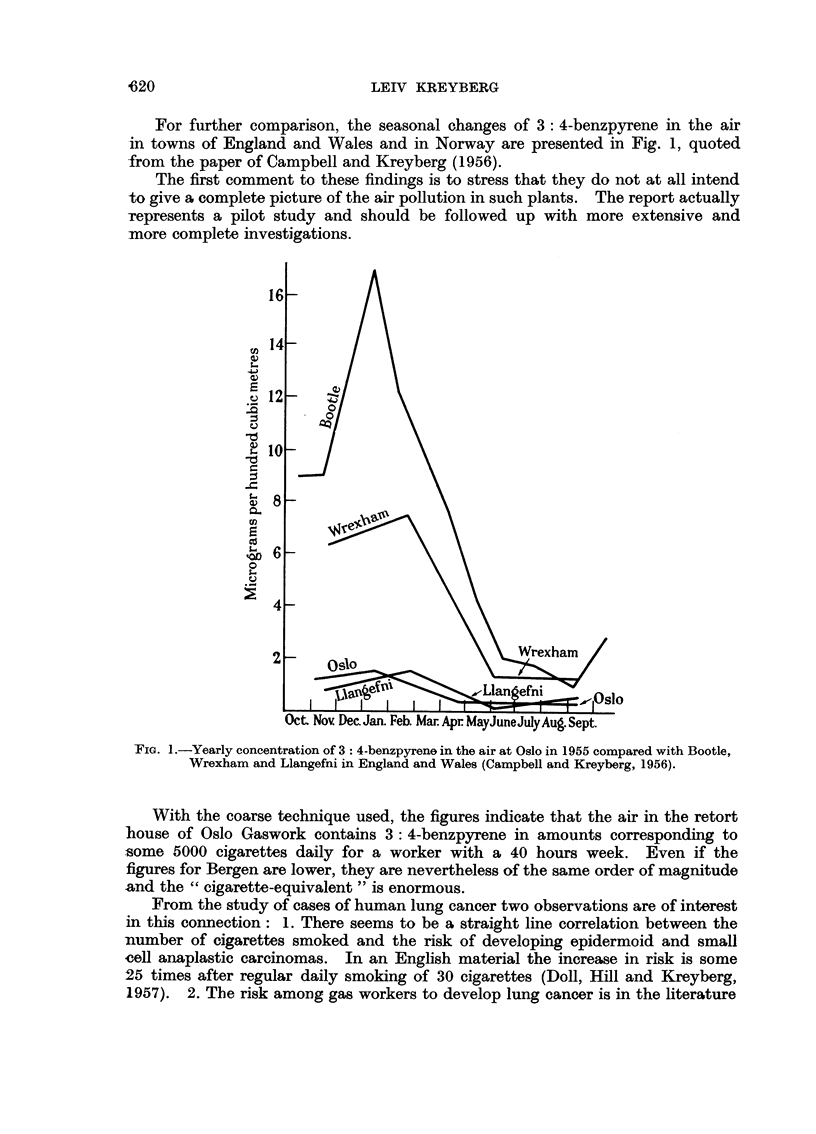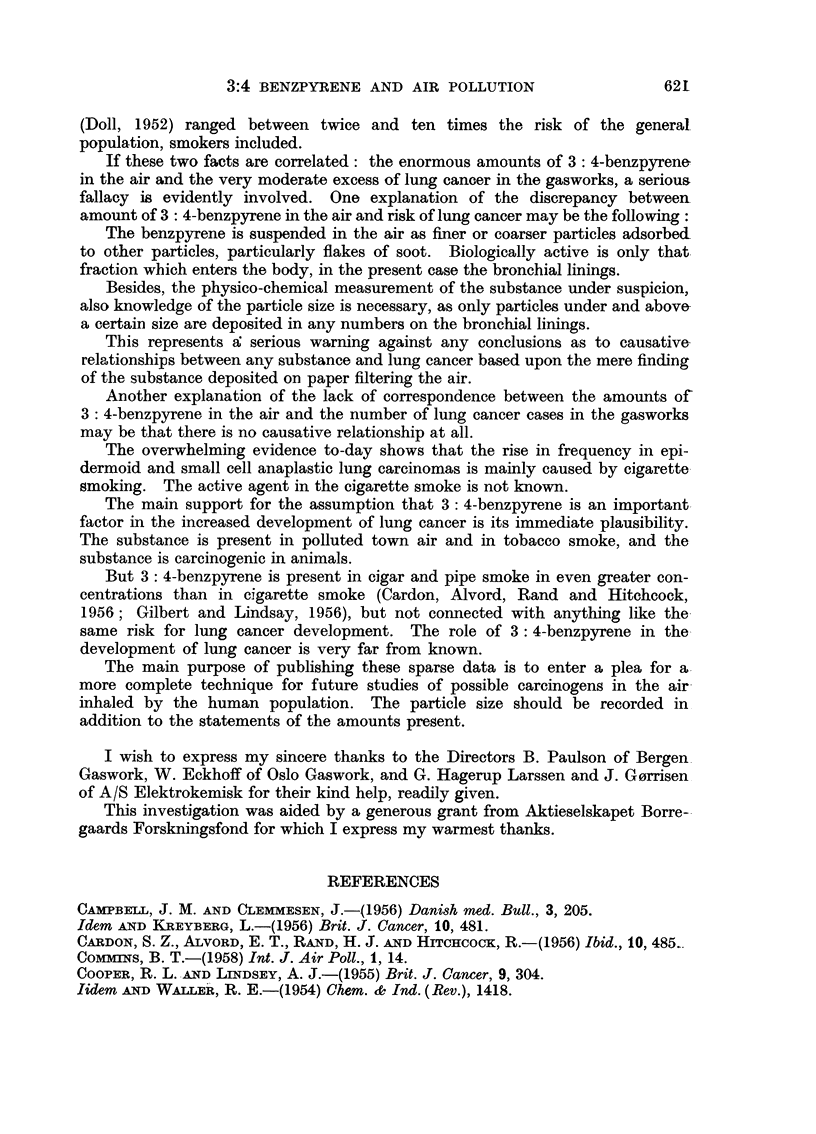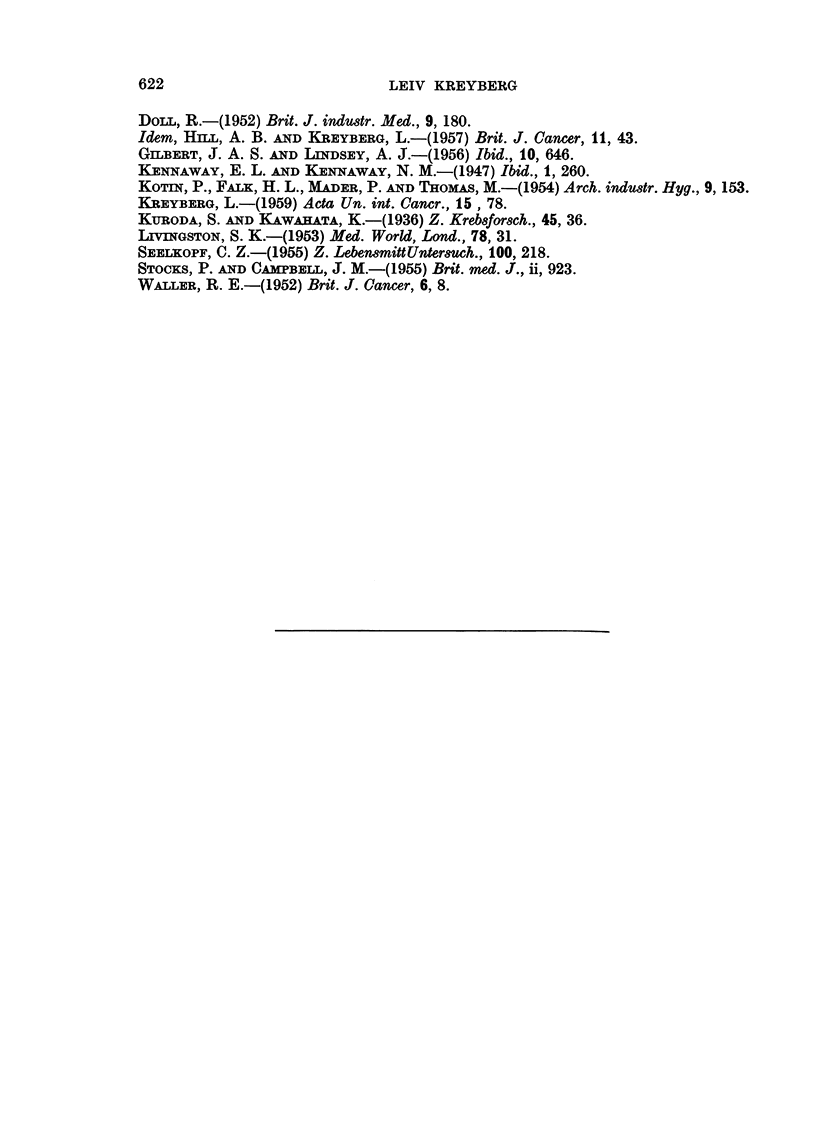# 3:4-Benzpyrene in Industrial Air Pollution: Some Reflexions

**DOI:** 10.1038/bjc.1959.67

**Published:** 1959-12

**Authors:** Leiv Kreyberg


### 3 : 4-BENZPYRENE IN INDUSTRIAL AIR POLLUTION: SOME REFLEXIONS

LEIV KREYBERG

*From the Institutt for Generell og Eksperimentell Patologi Universitetet i Oslo, Norway*

Received for publication July 30, 1959

AMONG the known carcinogens producing lung carcinoma in man: arsenic, asbestos, beryllium, chromium, nickel and ionizing agents can account for only a small fraction of all cases occurring, because these agents are very limited in their distributions.

As 3 : 4-benzpyrene, a well known experimental carcinogen for epidermis and connective tissue, has been found in tobacco smoke (Cooper, Lindsay and Waller, 1954; Cooper and Lindsay, 1955; Seelkopf, 1955) and in the air of populated areas, especially town air (Waller, 1952; Kotin, Falk, Mader and Thomas, 1954; Stocks and Campbell, 1955; Campbell and Clemmesen, 1956; Campbell and Kreyberg, 1956), the view has been advanced by numerous students, that 3 : 4-benzpyrene is the important factor in the increase in lung carcinomas in recent years, especially among males in towns.

Stocks and Campbell in their paper even attempted to calculate the respective role of 3 : 4-benzpyrene from the two sources. Such calculations are, however, of restricted interest because the cases of lung tumours used for the calculations were mainly based upon death certificates and the histological typing ignored. The latter point is of special importance when the absolute number of cases is low, as is the case in rural districts in Norway (Kreyberg, 1959). It may be of considerably lesser importance in England and Wales, where the lung cancer incidence is high, even in rural districts.

In the rural areas not only the amount of 3 : 4-benzpyrene in the general air is lower than in more densely populated areas and towns, but the number of special factories and industrial plants producing carcinogens are also fewer. A certain consideration should therefore be paid, when lung cancer frequency in urban and rural areas are compared, to the occupation of the victims.

It may be that some of the lung cancer patients in the rural districts have been exposed to important concentrations of carcinogens during their working hours, in spite of living in an air little polluted generally. With the increasing dispersion of industry in countries with ample access to water-power (and in the future to atomic power) an increasing population will work in factories with polluted air, in areas administratively designated as " rural ".

Under these circumstances a study of the occurrence of 3 : 4-benzpyrene in industrial plants should be of some interest.

Gasworks naturally present themselves for study, because gas fumes contain benzpyrene, and statistical evidence points in the direction that lung cancer occurs more frequently among gas workers, than in the general population (Kuroda and Kawahata, 1936; Kennaway and Kennaway, 1947; Doll, 1952).

Another source of air, polluted with 3 : 4-benzpyrene, came to my attention some years ago, when a paper by Livingston (1953) described warts appearing on the hands, arms, ankles and face of workers connected with the Søderberg furnace in an aluminium factory in England.

At the same time I had the opportunity to examine a tar developed during aluminium production in a Norwegian factory. The tar showed a very high carcinogenic potency when painted on the skin of mice, and by chemical analysis the tar was shown to contain 1 per cent 3 : 4-benzpyrene.

During the aluminium production fumes from such tars will in varying amount pollute the air of some parts of the factory.

On this background I sought contact with the gasworks of Bergen (director B. Paulson) and Oslo (director W. Eckhoff), as well as with the Norwegian State-owned and controlled aluminium factories (director Aa. W. Owe).

The two directors of the gasworks readily gave their full support. The director of the aluminium factories, however, flatly refused co-operation on similar terms. My application for permission to study the air in the privately owned A/S Elektrokemisk, which holds the rights of the Søderberg electrode was, however, immediately generously granted by the director-general G. Hagerup Larssen, and the director of Fiskaa Verk J. Gørrissen, and all facilities extended.

Gasmeters equipped with filter papers were installed in the plants at representative sites where the workers would be regularly employed, as described in a previous paper (Campbell and Kreyberg, 1956).

The filter papers were most kindly examined by Mr. B. T. Commins at the "Group for Research on Atmospheric Pollution", St. Bartholomew's Hospital, London.

The results of the investigations of the three industrial plants are summarized in Table I. For comparison figures from the air of some towns in England and Wales are given, quoted from Commins (1958).

TABLE I.—*The Content of Hydrocarbons in the Air in μg. per 100 m³*

| | Source of sample | | | | | |
|---|---|---|---|---|---|---|
| | Industrial Plants | | | General Town Air | | |
| Hydrocarbon | Oslo Gasworks | Bergen Gasworks | Elektrokemisk | Liverpool | Wrexham | Llangefni |
| | | | (μg. per 100 m³) | | | |
| Fluoranthene | 1300 | 240 | 100 | 6·7 | 2·0 | 0·4 |
| 1 : 2-benzanthracene | 1400 | 280 | 80 | .. | .. | .. |
| Phenanthrene | Not detected | 8 | 27 | .. | .. | .. |
| 1 : 2-benzpyrene | 150 | 130 | 10 | .. | .. | .. |
| Pyrene | 870 | 240 | 69 | 5·0 | 1·8 | 0·3 |
| Coronene | 20 | 30 | Trace | .. | .. | .. |
| 1 :12-benzperylene | 210 | 200 | 5 | 16·6 | 5·1 | 0·5 |
| **3 : 4-BENZPYRENE** | **730** | **200** | **18** | **6·8** | **2·0** | **0·4** |
| Anthracene | 100 | Trace | Present | .. | 0·4 | 0·1 |
| Anthrathene | 80 | 60 | Trace | .. | .. | .. |
| Fluorene | 20 | Trace | ,, | .. | .. | .. |
| Naphthalene | Not detected | Not detected | .. | .. | .. | .. |

For further comparison, the seasonal changes of 3 : 4-benzpyrene in the air in towns of England and Wales and in Norway are presented in Fig. 1, quoted from the paper of Campbell and Kreyberg (1956).

The first comment to these findings is to stress that they do not at all intend to give a complete picture of the air pollution in such plants. The report actually represents a pilot study and should be followed up with more extensive and more complete investigations.

Fig. 1.—Yearly concentration of 3 : 4-benzpyrene in the air at Oslo in 1955 compared with Bootle, Wrexham and Llangefni in England and Wales (Campbell and Kreyberg, 1956).

With the coarse technique used, the figures indicate that the air in the retort house of Oslo Gaswork contains 3 : 4-benzpyrene in amounts corresponding to some 5000 cigarettes daily for a worker with a 40 hours week. Even if the figures for Bergen are lower, they are nevertheless of the same order of magnitude and the " cigarette-equivalent " is enormous.

From the study of cases of human lung cancer two observations are of interest in this connection : 1. There seems to be a straight line correlation between the number of cigarettes smoked and the risk of developing epidermoid and small cell anaplastic carcinomas. In an English material the increase in risk is some 25 times after regular daily smoking of 30 cigarettes (Doll, Hill and Kreyberg, 1957). 2. The risk among gas workers to develop lung cancer is in the literature

(Doll, 1952) ranged between twice and ten times the risk of the general population, smokers included.

If these two facts are correlated : the enormous amounts of 3 : 4-benzpyrene in the air and the very moderate excess of lung cancer in the gasworks, a serious fallacy is evidently involved. One explanation of the discrepancy between amount of 3 : 4-benzpyrene in the air and risk of lung cancer may be the following :

The benzpyrene is suspended in the air as finer or coarser particles adsorbed to other particles, particularly flakes of soot. Biologically active is only that fraction which enters the body, in the present case the bronchial linings.

Besides, the physico-chemical measurement of the substance under suspicion, also knowledge of the particle size is necessary, as only particles under and above a certain size are deposited in any numbers on the bronchial linings.

This represents a serious warning against any conclusions as to causative relationships between any substance and lung cancer based upon the mere finding of the substance deposited on paper filtering the air.

Another explanation of the lack of correspondence between the amounts of 3 : 4-benzpyrene in the air and the number of lung cancer cases in the gasworks may be that there is no causative relationship at all.

The overwhelming evidence to-day shows that the rise in frequency in epidermoid and small cell anaplastic lung carcinomas is mainly caused by cigarette smoking. The active agent in the cigarette smoke is not known.

The main support for the assumption that 3 : 4-benzpyrene is an important factor in the increased development of lung cancer is its immediate plausibility. The substance is present in polluted town air and in tobacco smoke, and the substance is carcinogenic in animals.

But 3 : 4-benzpyrene is present in cigar and pipe smoke in even greater concentrations than in cigarette smoke (Cardon, Alvord, Rand and Hitchcock, 1956 ; Gilbert and Lindsay, 1956), but not connected with anything like the same risk for lung cancer development. The role of 3 : 4-benzpyrene in the development of lung cancer is very far from known.

The main purpose of publishing these sparse data is to enter a plea for a more complete technique for future studies of possible carcinogens in the air inhaled by the human population. The particle size should be recorded in addition to the statements of the amounts present.

I wish to express my sincere thanks to the Directors B. Paulson of Bergen Gaswork, W. Eckhoff of Oslo Gaswork, and G. Hagerup Larssen and J. Gørrisen of A/S Elektrokemisk for their kind help, readily given.

This investigation was aided by a generous grant from Aktieselskapet Borregaards Forskningsfond for which I express my warmest thanks.

### REFERENCES

Campbell, J. M. and Clemmesen, J.—(1956) *Danish med. Bull.*, **3**, 205.
*Idem* and Kreyberg, L.—(1956) *Brit. J. Cancer*, **10**, 481.
Cardon, S. Z., Alvord, E. T., Rand, H. J. and Hitchcock, R.—(1956) *Ibid.*, **10**, 485.
Commins, B. T.—(1958) *Int. J. Air Poll.*, **1**, 14.
Cooper, R. L. and Lindsey, A. J.—(1955) *Brit. J. Cancer*, **9**, 304.
*Iidem* and Waller, R. E.—(1954) *Chem. & Ind.* (*Rev.*), 1418.

DOLL, R.—(1952) *Brit. J. industr. Med.*, **9**, 180.
*Idem*, HILL, A. B. AND KREYBERG, L.—(1957) *Brit. J. Cancer*, **11**, 43.
GILBERT, J. A. S. AND LINDSEY, A. J.—(1956) *Ibid.*, **10**, 646.
KENNAWAY, E. L. AND KENNAWAY, N. M.—(1947) *Ibid.*, **1**, 260.
KOTIN, P., FALK, H. L., MADER, P. AND THOMAS, M.—(1954) *Arch. industr. Hyg.*, **9**, 153.
KREYBERG, L.—(1959) *Acta Un. int. Cancr.*, **15** , 78.
KURODA, S. AND KAWAHATA, K.—(1936) *Z. Krebsforsch.*, **45**, 36.
LIVINGSTON, S. K.—(1953) *Med. World, Lond.*, **78**, 31.
SEELKOPF, C. Z.—(1955) *Z. LebensmittUntersuch.*, **100**, 218.
STOCKS, P. AND CAMPBELL, J. M.—(1955) *Brit. med. J.*, ii, 923.
WALLER, R. E.—(1952) *Brit. J. Cancer*, **6**, 8.